# Assessment of lower limb muscle strength can predict fall risk in patients with chronic liver disease

**DOI:** 10.1038/s41598-023-50574-7

**Published:** 2024-01-02

**Authors:** Hitomi Takada, Koji Yamashita, Leona Osawa, Yasuyuki Komiyama, Masaru Muraoka, Yuichiro Suzuki, Mitsuaki Sato, Shoji Kobayashi, Takashi Yoshida, Shinichi Takano, Shinya Maekawa, Nobuyuki Enomoto

**Affiliations:** Gastroenterology and Hepatology Department of Internal Medicine, Faculty of Medicine, University of Yamanashi, Shimokato 1110, Chuo, Yamanashi 409-3898 Japan

**Keywords:** Gastroenterology, Risk factors, Signs and symptoms

## Abstract

Falls are caused by a combination of factors, including loss of lower limb muscle strength (LMS), and associated with declined performance status (PS). Age-related sarcopenia is generally associated with decreased muscle mass and strength of lower limb muscle but without a noticeable loss of those of upper limb or trunk muscle. However, no reports have focused on falls or LMS in chronic liver disease (CLD) patients. This study is the first to analyze the risk factors for falls in patients with CLD, focusing on LMS measurement using the Locomoscan. This study enrolled 315 CLD patients whose LMS was measured. The patients who experienced falls more than 1 year ago or during the observation period were classified as those who experienced falls. We found that risk factors for falls were PS1/2 and decreased LMS (< 0.32 N/kg). The group with sarcopenia had a higher frequency of decreased LMS (54 vs. 26%, *p* = 0.001) and falls (24 vs. 4.4%, *p* < 0.001) compared to the non-sarcopenia group. This study found that decreased LMS was an independent risk factor for falls. Assessment of LMS may be used as a better marker associated with the risk of falls in patients with CLD.

## Introduction

Falls are caused by a combination of factors, including loss of lower limb muscle strength (LMS), muscle mass, and balance, and frequently occur even in nonelderly patients with chronic diseases^1–3^. Over 33% of community-dwelling people aged above 65 years fall at least once per year, and 50% of them will experience recurrent falls. The frequency of falls may rise by as much as 60% as age increases^4,5^. Preventive measures include therapeutic intervention through nutrition, exercise and pharmacotherapy^6–8^. Therefore, prior diagnosis of patients with a high risk of falling and preventive therapeutic intervention may increase healthy life expectancy and reduce health care costs^9^.

Sarcopenia is characterized by the loss of muscle strength and skeletal muscle mass and has a poor prognosis in the elderly^10–13^. Age-related sarcopenia has been associated with shorter survival, fall fractures, infection, depression, and worsening performance status (PS)^14,15^. Secondary sarcopenia in patients with cancer or chronic diseases is also a poor prognostic factor. The incidence of sarcopenia in patients with chronic liver disease (CLD) has been increasing^12,16,17^. According to the guidelines on sarcopenia in liver disease by the Japan Society of Hepatology, sarcopenia is defined by decreased grip strength (GS) and skeletal muscle mass; it is a hot topic in the field of liver diseases^18^. Due to sarcopenia, the frequency of hospitalization in patients with primary hepatocellular carcinoma (HCC) increases as a result of fall fractures, infection, encephalopathy, ascites, and death^12^. Therefore, it is important to predict the risk of falls in patients with CLD, even if they are not elderly, as they have reduced muscle strength and muscle mass, i.e. sarcopenia is more frequent.

However, to the best of our knowledge, the risk of falls in patients with CLD has not yet investigated, and this prospective study is the first to analyze the risk factors for falls in patients with CLD, focusing on LMS measurement.

## Methods

### Patients

This study enrolled 315 patients with CLD who underwent medical examination at our hospital between September 2022 and August 2023 (Supplementary Fig. 1). The inclusion criteria were as follows: patients with CLD (with liver dysfunction noted on at least two blood tests or with blood abnormalities suggestive of liver cirrhosis, on medication for liver diseases, and treated for HCC), those with an Eastern Cooperative Oncology Group PS score of 0–2, and those aged over 18 years. The exclusion criteria were as follows: patients with inaccurate data (n = 18), those followed up for < 4 weeks (n = 2), those with paralysis (n = 1), those with a history of lower limb surgery (n = 8), those with body weight > 100 kg (n = 3), those with fall fracture episodes in the past year before the measurement (n = 5), and those with cancer of other organs (n = 2).

All patients provided written informed consent. This study was approved by the Human Ethics Review Committee of the University of Yamanashi (approval number: 1326) and performed in accordance with the Declaration of Helsinki.

### Diagnosis of sarcopenia

The Japan Society of Hepatology defines sarcopenia as decreased GS and low muscle mass in patients with CLD^18^. The GS of each hand was measured twice, and the mean value of the better of the two was adopted. According to the assessment criteria, the cutoff value for GS was 28 kg for men and 18 kg for women^18^.

Bioelectrical impedance analysis (BIA) or CT scan imaging was performed to evaluate the muscle mass. Using CT scan imaging, the psoas muscle mass index (PMI) at the third lumbar vertebra level indicated the muscle mass volume. CT scan images were taken 3 months before and after LMS measurement. PMI was determined by manually tracing the cross-sectional areas of the bilateral psoas muscles and normalizing the values to the square of the patient’s height in meters. Two hepatology specialists with expertise in this field measured the PMI and set the PMI cutoff value at 6.36 cm^2^/m^2^ for males and 3.92 cm^2^/m^2^ for females using the assessment criteria. Further, a registered dietitian with expertise in this field performed BIA using InBody 720 to calculate skeletal muscle mass. Skeletal muscle index (SMI) was calculated as the sum of muscle mass in the upper and lower extremities divided by height squared (kg/m^2^). The cutoff value for SMI was 7.0 kg/m^2^ for men and 5.7 kg/m^2^ for women, according to the assessment criteria.

CT values of the multifidus muscle at the level of the third lumbar vertebra were used as an indicator of skeletal muscle quality. The cutoff value for low CT values was identified as 44.4 Hounsfield Unit (HU) for males and 39.3 HU for females^17,19^.

### Measurement of lower limb muscle strength

The same examiners, two hepatologists and one nurse, measured LMS using the Locomoscan (AllCare Co. Ltd., Tokyo, Japan) and a dedicated assist frame^20,21^. In a sitting position on the examination bed, the patient placed the posterior knee joint on the pressure sensor of the Locomoscan and started the measurement after the ankle joint was fixed with the attached belt. The assist frame was set at 30° knee joint flexion and 0° ankle joint flexion. Before measurement, the patient was instructed to perform a knee extension exercise in which the posterior knee joint was pressed against the pressure sensor and the ankle belt was pulled upward. During measurement, the patient performed the exercise for 7 s under maximum effort, and two measurements were taken. Rest lasted for at least 30 s, until the patient’s fatigue improved. LMS was calculated as the mean value (N) of two left and right measurements divided by body weight (N/kg).

### Assessment of falls

A fall is an unplanned and unexpected descent to the ground, floor, or other lower level^22,23^. Patients or their accompanying family members were interviewed about the patients’ history of falls. Patients who experienced falls at least one year prior to the fall, i.e. who were considered to have lost the effects of temporary muscle weakness after the fall, or those experienced falls during the observation period (new falls) classified as those who experienced falls.

### Statistical analysis

All experimental data were expressed as median and range. The Mann–Whitney U test, Kruskal–Wallis test, and nonparametric analysis of variance were used to compare groups. Fisher’s exact test was used for group comparisons if the one-way analysis of variance was significant. The correlation of continuous variables was assessed using Spearman’s rank correlation coefficient. The statistical significance was set at a *p* value of < 0.05. All statistical analyses were performed using EZR (Saitama Medical Center, Jichi Medical University, Saitama, Japan), a graphical user interface for R (The R Foundation for Statistical Computing, Vienna, Austria)^24^. It is a modified version of the R commander designed to include statistical functions commonly used in biostatistics.

### Ethics approval statement, patient consent statement

Written informed consent was obtained from all patients, and this study was approved by the Human Ethics Review Committee of Yamanashi University Hospital (approval number: 1326), in accordance with the Declaration of Helsinki.

## Results

### Patient characteristics

The characteristics of patients with CLD are shown in Table 1. The median age was 67 (18–91), and 188 (60%) were male. CLD etiology was hepatitis B, hepatitis C, and non-B non-C hepatitis in 33, 85, and 197 patients, respectively. Of them, 98 were diagnosed with cirrhosis. Hepatic function was classified as Child–Pugh A and B in 298 and 17 patients, respectively, and serum albumin-bilirubin (ALBI) grade 1, 2a, 2b, and 3 in 247, 32, 28, and 8 patients.

**Table 1:** Patient characteristics.

	All (n = 315)	Male (n = 188)	Female (n = 127)	*p* value
Age, years old	67 (18–91)	67 (18–91)	68 (37–91)	0.47
Male, n	188 (60%)	–	–	–
Performance status (0/1 or 2), n	270/45 (86/14%)	167/21 (89/11%)	103/24 (81/19%)	0.070
Inability to cross a pedestrian crossing in time, n	11 (3.5%)	4 (2.1%)	7 (5.5%)	0.13
Body mass index	25 (15–44)	24 (15–44)	25 (17–39)	0.23
Decreased PMI or SMI, n	74 (27%)	55 (33%)	19 (18%)	0.008
PMI < men 6.36, women 3.92 cm^2^/m^2^, n	67 (28%)	51 (36%)	16 (16%)	0.001
SMI < men 7.0, women 5.7 kg/m^2^, n	31 (23%)	18 (22%)	13 (24%)	0.84
CT value < male 44.4, female 39.3 HU	126 (53%)	59 (42%)	67 (68%)	< 0.001
Grip strength < men 28, women 18 kg/m^2^, n	74 (23%)	40 (25%)	34 (30%)	0.50
Sarcopenia, n	41 (13%)	30 (16%)	11 (8.7%)	0.063
Etiology (HBV/HCV/nonBnonC), n	33/85/197 (10/27/63%)	22/57/109 (12/30/58%)	11/28/88 (9/22/69%)	0.13
Child–Pugh grade (A/B), n	298/17 (95/5%)	174/14 (93/7%)	124/3 (98/2%)	0.073
ALBI grade (1/2a/2b/3), n	247/32/28/8 (78/10/9/3%)	140/19/24/5 (75/10/13/2%)	107/13/4/3 (84/10/3/3%)	0.021
Liver cirrhosis, n	98 (31%)	65 (35%)	33 (26%)	0.11
Previous episodes of HCC, n	86 (27%)	71 (38%)	15 (12%)	< 0.001
Lower limb muscle strength, N/kg	0.42 (0.020–1.3)	0.45 (0.02–1.3)	0.36 (0.07–1.0)	0.003

### Muscle mass, CT values, and grip strength in patients with CLD

CT PMI, BIA SMI, and CT values were decreased in 67 (28%), 31 (23%), and 126 (53%) patients, respectively. Forty-one patients (13%) had sarcopenia.

**Figure 1:**
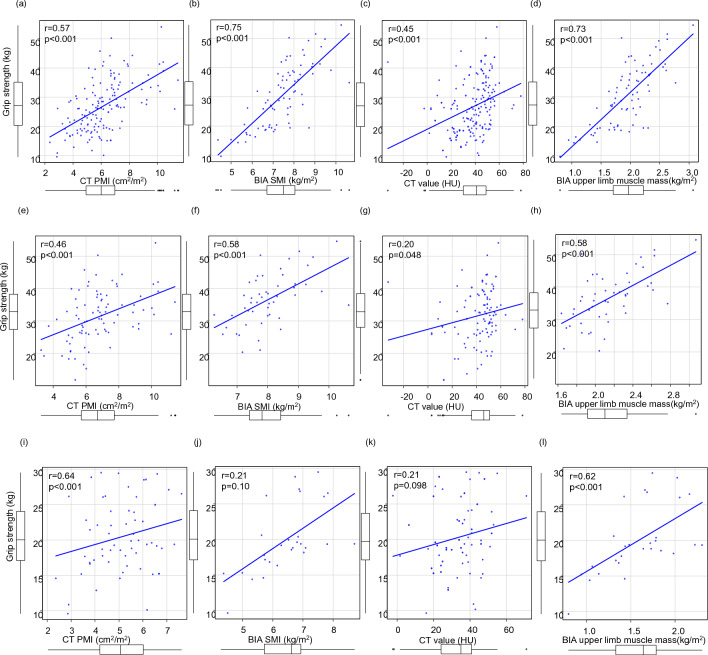
Correlation between grip strength (GS) and the other parameters in patients with chronic liver disease. (**a**) GS and psoas muscle mass index (PMI) using computed tomography (CT), (**b**) GS and skeletal muscle mass index (SMI) using bioelectrical impedance analysis (BIA), (**c**) GS and CT value of the multifidus muscle, (**d**) GS and upper limb muscle mass using BIA in all the patients. (**e**–**h**) In male patients. (**i**–**l**) in female patients.

GS was reduced in 74 (27%) patients and positively correlated with CT PMI (r = 0.57, *p* < 0.001), BIA SMI (r = 0.75, *p* < 0.001), CT value (r = 0.45, *p* < 0.001), and BIA upper limb muscle mass (r = 0.73, *p* < 0.001) in all patients (Fig. 1a–d). In particular, GS was strongly correlated to the upper limb muscle mass and BIA SMI, which reflects the total body muscle mass. A similar trend was observed in male patients (Fig. 1e–h). In contrast, in female patients, GS was positively correlated to BIA SMI and upper limb muscle mass, but not CT PMI and CT value (Fig. 1i–l).

### Lower limb muscle strength in patients with CLD

**Figure 2:**
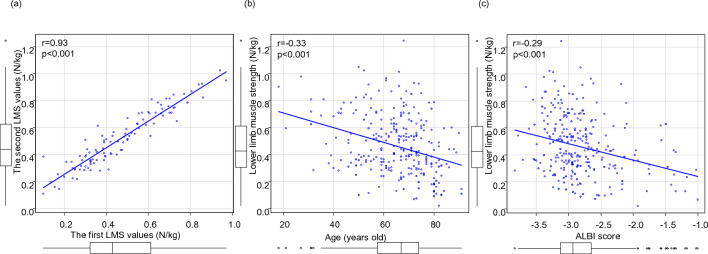
(**a**) Correlation between the first and second lower limb muscle strength (LMS) values. (**b**) Correlation between LMS and age, (**c**) Correlation between LMS and albumin-bilirubin (ALBI) score.

The first and second LMS values showed good correlation in the same patients using Locomoscan (r = 0.93, *p* < 0.001) (Fig. 2a). LMS worsened with increasing age (r = − 0.33, *p* < 0.001) and ALBI score (r = − 0.29, *p* < 0.001) and was similar for both male and female patients (Fig. 2b,c).

**Figure 3:**
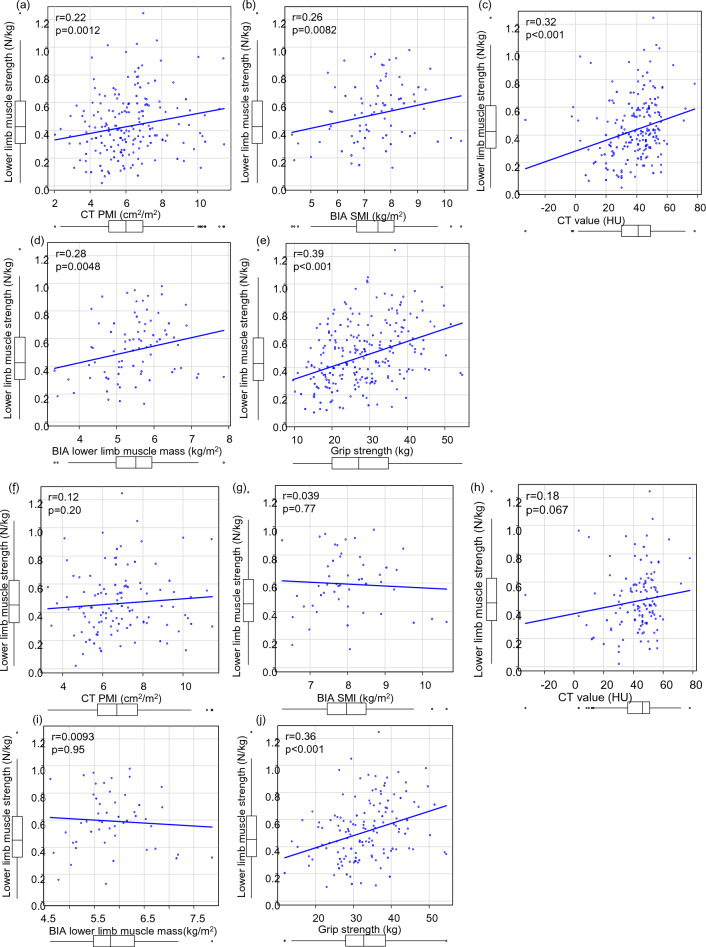

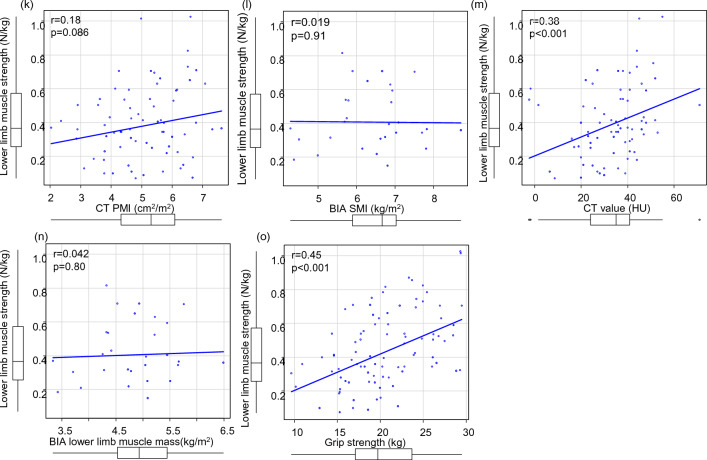
Correlation between lower limb muscle strength (LMS) and the other parameters in patients with chronic liver disease. (**a**) LMS and psoas muscle mass index (PMI) using computed tomography (CT), (**b**) LMS and skeletal muscle mass index (SMI) using bioelectrical impedance analysis (BIA), (**c**) LMS and CT value of the multifidus muscle, (**d**) LMS and lower limb muscle mass using BIA in all the patients, (**e**) LMS and grip strength (GS). (**f**–**j**) In male patients. (**k**–**o**) in female patients.

In all the patients, LMS was positively correlated with GS (r = 0.39, *p* < 0.001), CT PMI (r = 0.22, *p* = 0.012), BIA SMI (r = 0.26, *p* = 0.0082), CT value (r = 0.32, *p* < 0.001), and BIA lower limb muscle mass (r = 0.28, *p* = 0.0048) (Fig. 3a–e, Supplementary Table 1). In male patients, there was some correlation with GS, but not with other parameters. In contrast, for female patients, a positive correlation was found for GS and CT value, but not for CT PMI, BIA SMI, and BIA lower limb muscle mass (Fig. 3f–o).

### Risk factors for falls in patients with CLD

We found that 7.0% of CLD patients had experienced falls more than 1 year ago or during the observation period. We calculated the areas under the receiver operating characteristic (ROC) curves of LMS. The cutoff value of LMS for falls was 0.32 N/kg with ROC of 0.81 (0.71–0.91), sensitivity of 0.77, and specificity of 0.77. Patients who experienced falls (n = 22) had a higher frequency of lower LMS than those without falls (n = 293) (82% vs. 26%, *p* < 0.001) (Table 2). The group with sarcopenia had a higher frequency of falls (24 vs. 4.4%, *p* < 0.001) and decreased LMS (54 vs. 26%, *p* = 0.001) compared to the non-sarcopenia group.

**Table 2:** Comparison between the patients who experienced falls and those without falls.

	Patients who experienced falls (n = 22)	Patients without falls (n = 293)	*p* value
Age, years old	81 (64–91)	67 (18–91)	< 0.001
Male, n	11 (50%)	177 (60%)	0.37
Performance status (0/1 or 2), n	6/16 (27/73%)	264/29 (90/10%)	< 0.001
Inability to cross a pedestrian crossing in time, n	6 (27%)	5 (1.7%)	< 0.001
Body mass index	22 (17–27)	25 (15–44)	0.005
Decreased PMI or SMI, n	15 (68%)	59 (23%)	< 0.001
PMI < men 6.36, women 3.92 cm^2^/m^2^, n	15 (68%)	52 (24%)	< 0.001
SMI < men 7.0, women 5.7 kg/m^2^, n	13 (72%)	18 (15%)	< 0.001
CT value < male 44.4, female 39.3 HU	16 (73%)	110 (51%)	0.071
Grip strength < men 28, women 18 kg/m^2^, n	9 (53%)	65 (25%)	0.021
Sarcopenia, n	10 (46%)	31 (11%)	< 0.001
Etiology (HBV/HCV/nonBnonC), n	1/12/9 (5/54/41%)	32/73/188 (11/25/64%)	0.015
Child–Pugh grade (A/B), n	20/2 (91/9%)	278/15 (95/5%)	0.34
ALBI grade (1/2a/2b/3), n	13/3/4/2 (59/14/18/9%)	234/29/24/6 (80/10/8/2%)	0.034
Liver cirrhosis, n	11 (50%)	87 (30%)	0.057
Previous episodes of HCC, n	14 (64%)	72 (25%)	< 0.001
Lower limb muscle strength, N/kg	0.22 (0.020–0.56)	0.44 (0.070–1.3)	< 0.001

Risk factors for falls in patients with CLD in the univariate analysis were as follows: age > 73 years (odds ratio [OR] 14, *p* < 0.001), PS1/2 (OR 24, *p* < 0.001), previous episodes of HCC (OR 5.7, *p* < 0.001), ALBI grade 2b/3 (OR 3.3, *p* = 0.021), BMI < 20 (OR 4.8, *p* = 0.0018), decreased GS (OR 3.3, *p* = 0.017), decreased muscle mass in CT/BIA (OR 7.1, *p* < 0.001), inability to cross a pedestrian crossing in time (OR 18, *p* < 0.001), and decreased LMS (OR 13, *p* < 0.001) (Table 3). For the multivariate analysis, PS1/2 (OR 9.7, *p* = 0.0069) and decreased LMS (OR 17, *p* = 0.0028) were independent factors.

**Table 3:** Risk factors for falls in patients with chronic liver disease.

	Univariate analysis		Multivariate analysis	
	OR (range)	*p* value	OR (range)	*p* value
Age > 73 years old	14 (4.7–44)	< 0.001	3.2 (0.48–21)	0.23
Male	0.66 (0.28–1.6)	0.34		
Performance status 1/2	24 (8.8–67)	< 0.001	9.7 (1.9–50)	0.0069
Inability to cross a pedestrian crossing in time	18 (4.7–69)	< 0.001	3.5 (0.32–39)	0.30
Body mass index < 20	4.8 (1.8–13)	0.0018	2.3 (0.33–16)	0.40
Decreased PMI or SMI	7.1 (2.7–18)	< 0.001	4.9 (0.88–27)	0.069
PMI < men 6.36, women 3.92 cm^2^/m^2^	6.8 (2.7–18)	< 0.001		
SMI < men 7.0, women 5.7 kg/m^2^	14 (4.6–46)	< 0.001		
CT value < male 44.4, female 39.3 HU	2.6 (0.99–6.9)	0.053		
Grip strength < men 28, women 18 kg/m^2^	3.3 (1.2–9.0)	0.017	1.3 (0.30–5.9)	0.71
Etiology: HCV vs. HBV	5.3 (0.66–42)	0.12		
nonBnonC vs. HBV	1.5 (0.19–13)	0.69		
Child–Pugh grade B	1.9 (0.40–8.7)	0.43		
ALBI grade 2b/3	3.3 (1.2–9.0)	0.021	2.9 (0.39–22)	0.30
Liver cirrhosis	2.4 (0.99–5.7)	0.053		
Previous episodes of HCC	5.7 (2.2–13)	< 0.001	1.1 (0.14–5.6))	0.90
Lower limb muscle strength < 0.32 N/kg	13 (4.2–39)	< 0.001	17 (2.7–109)	0.0028

In addition, we re-grouped the patients into two groups using age > 73 years, one of the risk factors for falls. Decreased LMS was an independent risk factor for falls in the group aged 73 years and over (OR 18, *p* = 0.0046), whereas there was only a trend in the group aged under 73 years (OR 3.9, *p* = 0.084) (Supplementary Table 2).

### Characteristics of the group with decreased lower limb muscle strength

The patients with decreased LMS (n = 94, 30%) were more likely to be over 73 years old, and had with LC, PS1/2, previous episodes of HCC, Child–Pugh grade B, ALBI grade 2b/3, decreased GS, decreased muscle mass in CT/BIA, sarcopenia, and inability to cross a pedestrian crossing in time, compared to the group without decreased LMS (Table 4).

**Table 4:** Comparison between the patients with decreased lower limb muscle strength and those without.

	Patients with decreased LMS (n = 94)	Patients without decreased LMS (n = 221)	*p* value
Age, years old	73 (32–91)	65 (18–90)	< 0.001
Male, n	46 (49%)	142 (64%)	0.012
Performance status 1/2, n	29 (31%)	16 (7.0%)	< 0.001
Inability to cross a pedestrian crossing in time, n	7 (7.4%)	4 (1.8%)	0.019
Body mass index < 20	11 (12%)	22 (10%)	0.69
Decreased PMI or SMI, n	31 (37%)	43 (27%)	0.019
PMI < men 6.36, women 3.92 cm^2^/m^2^, n	27 (34%)	40 (25%)	0.17
SMI < men 7.0, women 5.7 kg/m^2^, n	19 (45%)	12 (13%)	< 0.001
CT value < male 44.4, female 39.3 HU	53 (67%)	73 (45%)	0.002
Grip strength < men 28, women 18 kg/m^2^, n	33 (42%)	41 (25%)	< 0.001
Sarcopenia, n	22 (23%)	19 (8.6%)	0.001
Etiology (HBV/HCV/nonBnonC), n	8/32/54 (9/34/57/%)	25/53/143 (11/24/65%)	0.18
Child–Pugh grade (A/B), n	84/10 (89/11%)	214/7 (97/3%)	0.012
ALBI grade (1/2a/2b/3), n	61/14/13/6 (65/15/14/6%)	186/18/15/2 (84/8/7/1%)	0.001
Liver cirrhosis, n	43 (45%)	56 (25%)	0.001
Previous episodes of HCC, n	39 (42%)	47 (21%)	< 0.001
With falls, n	18 (19%)	4 (1.8%)	< 0.001

### Risk factors for falls by gender

For the univariate analysis of male patients, the following were significant: age > 73, PS1/2, previous episodes of HCC, BMI < 22, decreased GS, decreased muscle mass in CT/BIA, and decreased LMS. In the multivariate analysis, decreased muscle mass in CT/BIA (OR 13, *p* = 0.021) and decreased LMS (OR 5.0, *p* = 0.043) were independent factors (Table 5).

**Table 5:** Risk factors for falls by gender.

	Univariate analysis		Multivariate analysis	
	OR (range)	*p* value	OR (range)	*p* value
Male (n = 188)				
Age > 73 years old	5.5 (1.5–20)	0.0092	1.1 (0.11–6.8)	0.90
Performance status 1/2	13 (3.5–48)	< 0.001	4.4 (0.67–29)	0.12
Body mass index < 20	5.4 (1.4–20)	0.013	1.8 (0.30–11)	0.53
Decreased PMI or SMI	25 (3.1–200)	0.0025	13 (1.5–111)	0.021
Grip strength < men 28, women 18 kg/m^2^	11 (2.0–54)	0.0051	3.6 (0.56–23)	0.18
Previous episodes of HCC	8.4 (1.8–40)	0.0078	1.8 (0.20–16)	0.61
Lower limb muscle strength < 0.32 N/kg	6.2 (1.7–22)	< 0.001	5.0 (1.1–24)	0.043
Female (n = 127)				
Age > 79 years old	54 (10–286)	0.0016	7.1 (0.73–68)	0.091
Performance status 1/2	73 (8.7–613)	< 0.001	13 (0.51–305)	0.12
Inability to cross a pedestrian crossing in time	48 (7.6–297)	< 0.001	19 (1.1–327)	0.043
Decreased PMI or SMI	4.9 (1.3–18)	0.0018	15 (0.99–235)	0.051
Previous episodes of HCC	8.8 (2.3–34)	< 0.001	2.6 (0.028–5.3)	0.48
Lower limb muscle strength < 0.32 N/kg	9.6 (2.0–47)	0.0050	8.2 (0.79–84)	0.077

For the univariate analysis of female patients, the following were significant: age > 79, PS1/2, previous episodes of HCC, decreased muscle mass in CT/BIA, inability to cross a pedestrian crossing in time, and decreased LMS. In the multivariate analysis, the inability to cross the pedestrian crossing in time (OR 19, *p* = 0.043) was an independent factor (Table 5).

## Discussion

Falls were found in 7.0% of patients with CLD, and decreased LMS, an independent risk factor for falls, was found in 30%. This study examined the association between LMS and falls in patients with CLD. Traditional parameters for sarcopenia in patients with CLD, such as muscle mass and GS measurements alone, may not adequately identify high-risk groups for falls. Moreover, patients with low LMS should be followed up with particular attention to the risk of falls.

Sarcopenia is characterized by low skeletal muscle mass, skeletal muscle weakness, and decreased physical performance^10^. It causes a decline in functional status, impaired mobility, a higher risk of falls, and an increased mortality risk^25^. The prevalence of sarcopenia is reported as 10% in the general elderly population and 39% in cancer patients^26–28^. Skeletal muscle mass decreases by 1% per year, so by age 50, there is a 5–10% decrease compared to age 20, and after age 50, there is a 30–40% decrease^29^. Furthermore, muscle strength declines by 2–5% per year, and the rate of decline increase after the age 50^30,31^. Sarcopenia, which increases with age, was associated with falls, fractures, functional decline, and all-cause mortality in elderly people^32^. In contrast, sarcopenia is also considered as a poor prognostic factor in patients with CLD, even if they are not elderly, and is associated with hospitalization events in patients with primary HCC, including fall fractures^12,13,18^. Therefore, sarcopenia has received increasing attention in recent years among patients with CLD.

Falls are associated with declined PS, poor quality of life, and death in the elderly and patients with cirrhosis. In particular, the incidence of falls was 40% per year in those with minimal hepatic encephalopathy compared with 13% in those without^33,34^. In addition, fall trauma occurs in 54–74% and fractures in 6–12% of cases^35–37^. Fall accidents have serious mortality consequences. In this study, falls occurred in 7.0% of patients with CLD, despite the fact that many of these patients were young and had good liver function. Preventing falls, which necessitate nursing care and becoming bedridden, may be vital for enhancing healthy life expectancy in patients with CLD.

Few reports have assessed LMS, and the gold standard diagnostic method for decreased LMS and its cutoff values are still unknown. However, falls are caused by a combination of declined LMS, low muscle mass, and imbalance^36,38^. Hence, the decrease in LMS must be accurately assessed. Large multifunctional muscle strength measuring machines used for LMS measurement require a labor-intensive operation, and in clinical settings, measurements are rarely performed, even if the machines are available in the facility. However, due to the complexity and risk of falling during measurement, LMS measurement using simple devices such as handheld dynamometers has not been widely used^39^. In addition, age-related sarcopenia is generally associated with a loss of lower limb muscle mass and LMS but without a noticeable loss of upper limb or trunk muscle mass. The Locomoscan used in this study is an important noninvasive device that solves several LMS measurement problems. Omori et al. reported a high correlation coefficient of 0.82 between the quadriceps trainer, a prototype of the Locomoscan, and knee extension force measurements using the large multifunctional muscle strength measuring machines in healthy populations^21^. Therefore, this study focused on LMS measurement using the Locomoscan to indicate groups at high risk for falls.

This study found that LMS did not correlate strongly with CT PMI, BIA SMI, or regional muscle mass using BIA, unlike GS. Decreased LMS may capture earlier muscle weakness better than the other parameters. The LMS measurement requires more integrated capability, such as instantaneous force and balance, compared to GS, and no direct relationship may have been found. The results also suggest that LMS may not correlate as well as lower limb muscle mass due to overestimation of BIA caused by slight edema in patients with CLD. Moreover, the fact that local muscle mass and quality is hardly related to lower GS/LMS in female patients suggests that small number of cases in this study, the different areas prone to muscle changes depending on gender, and the possibility of other causes of lower GS/LMS; such as instantaneous force and balance are related. Thus, we believe that assessments related to muscle strength, such as LMS, in addition to the assessment of muscle quantity and quality using imaging studies are important as clinically useful markers.

In this study, the risk factors for falls in patients with CLD were PS1/2 and decreased LMS. Moreover, LMS was decreased in 64% of PS1/2 patients. Of the 270 PS0 patients, 65 (24%) had decreased LMS. This indicates that imaging alone cannot accurately predict patients at high risk for falls and that careful muscle strength measurements are important. It is particularly noteworthy that older age was not an independent factor. We believe that muscle assessment in all patients with CLD, regardless of age, is important for improving prognosis. LMS measurement may be useful to provide therapeutic intervention for fall risk before progression to PS1/2.

This study had some limitations. First, this study evaluated a small number of cases over a short time. Large-scale long-term studies are necessary. Second, we did not assess balance deficits. Reported fall risk factors include muscle weakness and balance deficits^40^. Balance is important for maintaining postural equilibrium and, thus, avoiding falls. Furthermore, balance exercises help older adults counteract balance deficits and reduce the risk of falls. Balance deficits are noteworthy in patients with ascites or hepatic encephalopathy and require further investigation. Third, no data are available on the therapies for sarcopenia. For elderly populations, supplemental vitamin D3, high protein diets, and resistance exercise training can help improve muscle mass and functions. Drug therapies such as testosterone and myostatin antibodies might potentially affect sarcopenia treatment. Additional research on this subject is required.

This study found a decreased LMS in 30% of patients with CLD, a high-risk group for falls. The group with sarcopenia had a higher frequency of decreased LMS compared to the non-sarcopenia group. Assessment of LMS may be used as a better marker associated with the risk of falls than sarcopenia, and may improve the prognosis of such patients.

## Conclusion

This study found that 7.0% of patients with CLD had experienced falls. Patients with falls had a higher frequency of lower LMS than those without, and decreased LMS was an independent risk factor for falls. Assessment of LMS may be used as a simple marker associated with the risk of falls in patients with CLD.

### Supplementary Information

Supplementary Figure 1.

Supplementary Tables.

## Data Availability

The datasets used during the current study available from the corresponding author on reasonable request.
