# Supplementary figures and images for: Assessment of lower limb muscle strength can predict fall risk in patients with chronic liver disease
Source: Sci Rep. 2024 Jan 2;14:64. doi: 10.1038/s41598-023-50574-7 (PMC10761732; doi:10.1038/s41598-023-50574-7)

Supplementary Fig 1. Flowchart of patient enrollment.

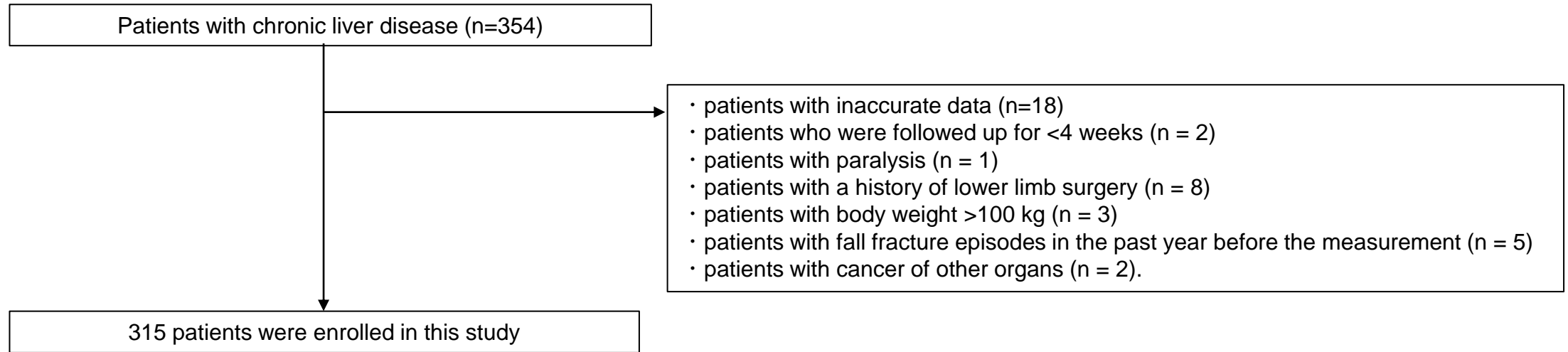

Supplement: Supplementary file 1 — Supplementary Figure 1. [file 41598_2023_50574_MOESM1_ESM.pdf]
