# Supplementary material for: Assessment of lower limb muscle strength can predict fall risk in patients with chronic liver disease
Source: Sci Rep. 2024 Jan 2;14:64. doi: 10.1038/s41598-023-50574-7 (PMC10761732; doi:10.1038/s41598-023-50574-7)
Supplement: Supplementary file 2 — Supplementary Tables. [file 41598_2023_50574_MOESM2_ESM.docx]

Supplementary Table 1. Variables affecting LMS values

| Variables | Coefficients | Standard errors | t-value | p-value | Lower limit 95% | Upper limit 95% |
| --- | --- | --- | --- | --- | --- | --- |
| age | -0.068 | -0.14 | -1.9 | 0.058 | 0.0024 | 0.035 |
| Male | 0.11 | 0.033 | 3.3 | 0.0011 | 0.044 | 0.18 |
| Performance status | -0.033 | 0.051 | -0.65 | 0.52 | -0.13 | 0.067 |
| Inability to cross a pedestrian crossing in time | -0.023 | 0.084 | -0.27 | 0.79 | -0.19 | 0.14 |
| Decreased PMI or SMI | -0.032 | 0.035 | -0.91 | 0.36 | -0.10 | 0.037 |
| CT value | -0.069 | 0.031 | -2.2 | 0.028 | -0.13 | -0.0075 |
| Grip strength | -0.065 | 0.033 | -1.9 | 0.053 | -0.13 | 0.00084 |
| Child-Pugh grade | -0.19 | 0.071 | -2.7 | 0.0075 | -0.33 | -0.052 |
| Liver cirrhosis | 0.014 | 0.036 | 0.39 | 0.70 | -0.057 | 0.084 |
| Previous episodes of HCC | -0.074 | 0.039 | -1.9 | 0.057 | -0.15 | 0.0022 |

Supplementary Table 2. Risk factors for falls in patients aged 73 years and over and those aged under 73 years.

|  | Univariate analysis |  | Multivariate analysis |  |
| --- | --- | --- | --- | --- |
|  | OR (range) | p value | OR (range) | p value |
| The group aged 73 years and over (n=88) |  |  |  |  |
| Male | 0.40 (0.14-1.2) | 0.091 |  |  |
| Performance status 1/2 | 13 (3.5-52) | <0.001 | 10 (2.0-51) | 0.0049 |
| Inability to cross a pedestrian crossing in time | 11 (2.5-51) | 0.0018 | 4.3 (0.56-33) | 0.16 |
| Body mass index < 20 | 3.9 (1.1-13) | 0.030 | 7.2 (1.1-45) | 0.035 |
| Decreased PMI or SMI | 2.4 (0.83-7.0) | 0.11 |  |  |
| CT value < male 44.4, female 39.3 HU | 1.0 (0.31-3.2) | 0.99 |  |  |
| Grip strength < men 28, women 18 kg/m^2^ | 1.2 (0.36-3.7) | 0.82 |  |  |
| Etiology: HCV vs. HBV | 1.3 (0.12-13) | 0.84 |  |  |
| nonBnonC vs. HBV | 0.88 (0.087-8.8) | 0.91 |  |  |
| Child-Pugh grade B | 1.3 (0.13-13) | 0.82 |  |  |
| ALBI grade 2b/3 | 2.3 (0.68-7.9) | 0.18 |  |  |
| Liver cirrhosis | 2.6 (0.89-7.3) | 0.082 |  |  |
| Previous episodes of HCC | 2.4 (0.80-7.0) | 0.12 |  |  |
| Lower limb muscle strength < 0.32 N/kg | 11 (2.3-50) | 0.0027 | 18 (2.4-135) | 0.0046 |
| The group aged under 73 years (n=227) |  |  |  |  |
| Male | 2.6 (0-Inf) | 0.99 |  |  |
| Performance status 1/2 | 7.1 (0.68-75) | 0.10 |  |  |
| Inability to cross a pedestrian crossing in time | 0.0013 (0-Inf) | 0.99 |  |  |
| Body mass index < 20 | 3.8 (0.38-38) | 0.26 |  |  |
| Decreased PMI or SMI | 2.8 (0-Inf) | 0.99 |  |  |
| CT value < male 44.4, female 39.3 HU | 4.3 (0.44-42) | 0.21 |  |  |
| Grip strength < men 28, women 18 kg/m^2^ | 8.5 (0.75-97) | 0.083 |  |  |
| Etiology: HCV vs. HBV | 1.9 (0-Inf) | 0.99 |  |  |
| nonBnonC vs. HBV | 1.0 (0-Inf) | 1.0 |  |  |
| Child-Pugh grade B | 5.9 (0.57-61) | 0.14 |  |  |
| ALBI grade 2b/3 | 3.4 (0.34-34) | 0.30 |  |  |
| Liver cirrhosis | 1.2 (0.084-8.1) | 0.87 |  |  |
| Previous episodes of HCC | 4.6 (0.63-34) | 0.13 |  |  |
| Lower limb muscle strength < 0.32 N/kg | 3.9 (0.53-28) | 0.084 |  |  |
